# Biosynthesis of Chlorophyll and Other Isoprenoids in the Plastid of Red Grape Berry Skins

**DOI:** 10.1021/acs.jafc.2c07207

**Published:** 2023-01-18

**Authors:** António Teixeira, Henrique Noronha, Sarah Frusciante, Gianfranco Diretto, Hernâni Gerós

**Affiliations:** † Centre of Molecular and Environmental Biology, Department of Biology, University of Minho, 4710-057 Braga, Portugal; ‡ Italian National Agency for New Technologies, Energy and Sustainable Development (ENEA), Casaccia Research Centre, 00123 Rome, Italy

**Keywords:** grape berry skins, plastid isolation, freeze dry, HPLC-MS analysis

## Abstract

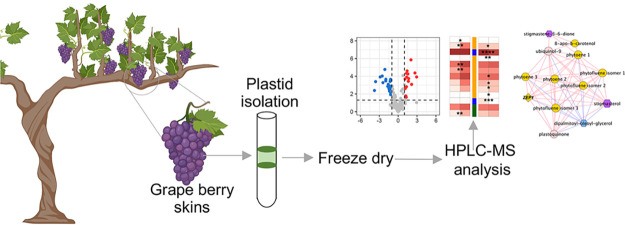

Despite current knowledge showing that fruits like tomato and grape berries accumulate different components of the light reactions and Calvin cycle, the role of green tissues in fruits is not yet fully understood. In mature tomato fruits, chlorophylls are degraded and replaced by carotenoids through the conversion of chloroplasts in chromoplasts, while in red grape berries, chloroplasts persist at maturity and chlorophylls are masked by anthocyanins. To study isoprenoid and lipid metabolism in grape skin chloroplasts, metabolites of enriched organelle fractions were analyzed by high-performance liquid chromatography-high-resolution mass spectrometry (HPLC-HRMS) and the expression of key genes was evaluated by real-time polymerase chain reaction (PCR) in berry skins and leaves. Overall, the results indicated that chloroplasts of the grape berry skins, as with leaf chloroplasts, share conserved mechanisms of synthesis (and degradation) of important components of the photosynthetic machinery. Some of these components, such as chlorophylls and their precursors, and catabolites, carotenoids, quinones, and lipids have important roles in grape and wine sensory characteristics.

## 1 Introduction

Photosynthesis in fruits like coffee, pea, soybean, avocado, orange, apple, tomato, cucumber, or grape berry is still puzzling as fruits are typically sink tissues. It has been proposed that the green tissues of fruits may contribute to carbon accumulation, providing O_2_ for respiration in the inner tissues, or even energy for metabolism and cell maintenance.^1−5^ Indeed, early studies showed that tomato fruit photosynthesis contributes to 10–15% of sugars accumulated in the fruit.^6,7^ More recently, proteomic analysis demonstrated that all components of the Calvin cycle, photorespiratory cycle, and the major light-harvesting proteins accumulate in tomato fruit at both mature green and breaker stages.^8^ Moreover, a recent study on grape berries cv. “Vinhão” revealed different proteins of the light reactions significantly accumulated in chloroplasts at the mature stage, suggesting that energy-yielding reactions predominate at this stage, while chloroplasts at the green stage accumulate proteins involved in the Calvin cycle.^5^ In tomato fruit, photosynthesis may rely exclusively on the CO_2_ released from internal respiration,^9^ due to the absence of stomata.^4^ Much like in tomato, the net uptake of CO_2_ by grape berries is likely very low because, depending on the cultivar, young fruits^10−12^ have few stomata, and these become wax-filled during maturation.^13^ Thus, in mature berries, the absence of stomata and the development of a waxy cuticle result in an internal environment with low O_2_ and high CO_2_ levels.^11^ This hypoxic environment may lead to cell death and fruit damage at higher temperatures, when respiration is stimulated; this is a serious problem that is becoming more frequent in the context of ongoing global warming.^14^

A distinct tissue-specific distribution pattern of photosynthetic competence was observed in the white grape berries from cv. Alvarinho.^1^ Indeed, the exocarp revealed the highest photosynthetic capacity and the lowest susceptibility to photoinhibition, while low fluorescence signals and photochemical competence were found in the mesocarp. Subsequent studies in the same variety showed that the photosynthetic activity of the exocarp was responsive to low- and high-light microclimate intensity differences in the canopy.^15^ More recently, we confirmed that the number of chloroplasts (and chlorophyll amount) in the pigmented skins of mature red grape berries is equivalent to their abundance at the green stage,^5^ which contrasts with tomato, where chlorophylls are replaced by carotenoids during ripening, accounting for its color change during maturation.^16^

Photosynthesis is the main contributor of redox equivalents and energy fueling biosynthesis of carbohydrates, amino acids, and secondary metabolites, such as isoprenoids synthesized through the mevalonate-independent pathway. These include photosynthesis-related pigments such as carotenoids and the phytol moiety of chlorophylls, and the side chain of electron carriers such as plastoquinones, phylloquinone, tocopherols, and hormones such as gibberellins and abscisic acid.^17,18^ In this regard, chloroplasts in berry skin are likely to play important roles in fruit metabolism and sensory characteristics. The major bottleneck for carotenoid biosynthesis is mediated by phytoene synthase encoded by *VvPSY*.^19^ In turn, at the end of the pathway, *VvNCEDs* code for 9-*cis*-epoxycarotenoid dioxygenase, which is involved in the biosynthesis of key abscisic acid (ABA) precursors.^20^ Furthermore, plastids are also one of the major compartments for the biosynthesis of several lipids, which are important structural and metabolic constituents of plant/fruit cells: in fact, acetyl-CoA, which is a direct product of photosynthesis, is a precursor of fatty acids and plastid membrane lipids.^21,22^ The enzyme acetyl-CoA carboxylase (ACCase), encoded by *VvACCase*, catalyzes the irreversible carboxylation of acetyl-CoA to malonyl-CoA.^23^

In mature leaf cells, the majority of lipids are localized in chloroplasts^24^ and occur mainly as galactolipids of thylakoid membranes and as a mixture of triacylglycerols (TAG), free fatty acids, and other lipophilic substances accumulated in plastoglobules, small lipid-rich droplets associated with thylakoid membranes.^21^ Along with structural roles, fatty acids and lipids often serve as precursors of important regulatory molecules (e.g., jasmonates), cuticle waxes, and aroma volatile substances.^25^ Indeed, grape berry lipids are precursors of the synthesis of wine aroma compounds, including volatile fatty acids.^26^ Specifically, different lipids, including stearic, palmitic, lauric, and myristic acids, are accumulated more in the grape berry at mature stage, while leaves are richer in stearic, palmitic, and lauric acids than grape berries.^27^ Of note, a tight relationship between wax’s triterpenoid components on the fruit surface and microbiome communities was recently established.^28^

In the present study, we hypothesize that the chloroplasts of berry skins are actively involved in the biosynthesis of isoprenoids and lipids, including chlorophylls, carotenoids, and precursors of ripening-related pathways. To address this hypothesis, plastids from green and mature grape exocarps and leaves (E-L 34 and E-L 38) were isolated from cv. “Vinhão” for metabolomic analysis. In parallel, the expression of key isoprenoid genes was evaluated by real-time polymerase chain reaction (PCR) in berry skins and leaves. Results highlighted the contribution of berry skin plastids to the metabolism of isoprenoids and lipids, some of which are essential for grape berry development and quality traits. Also, results suggest new avenues of research about the contribution of green berry tissues in the synthesis of important compounds for maturation and response to the environment. These include aromatic amino acids (synthesis of anthocyanins), precursors of key volatiles, and ABA. Another opportunity for future research is the role of skin plastids in other agriculturally important fleshy fruits, like the tomato, which accumulates lycopene at the mature stage.

## 2 Materials and Methods

### 2.1 Plant Material

**Figure 1:**
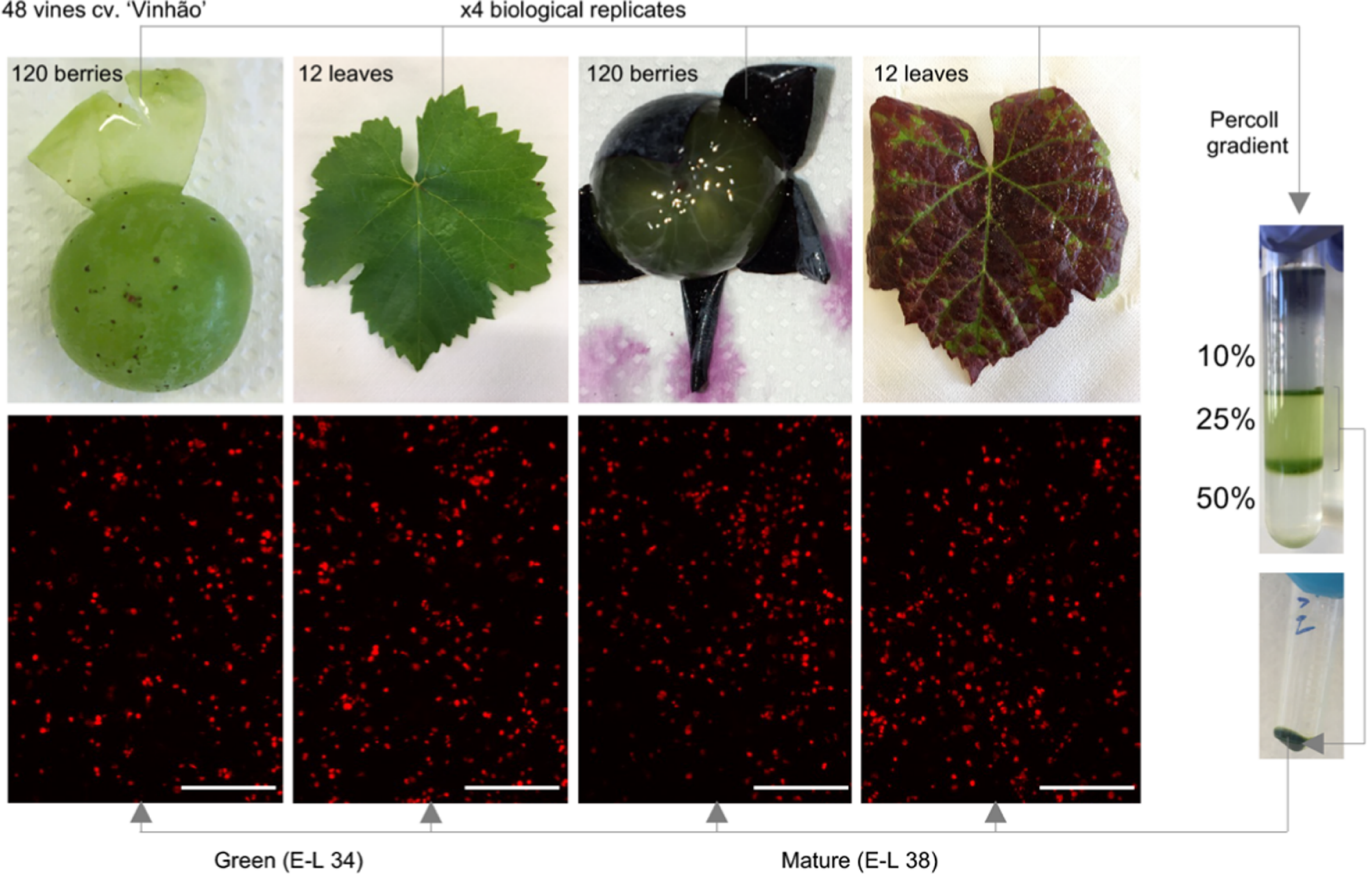
Plastid purification from leaves and grape berry skins of Vitis vinifera cv. “Vinhão” at green and mature developmental stages. Chloroplast samples were observed under an epifluorescence microscope.

Grape berries and leaves from the red cultivar “Vinhão” (*Vitis vinifera*) were sampled in the 2020 season in a Portuguese ampelographic collection (Estação Vitivinícola Amândio Galhano, N 41° 48′ 55.55″/W 8° 24′ 38.07″), located in the Controlled Appellation (DOC) region of Vinhos Verdes in the northwest region of Portugal from 33-year-old vines trained and spur-pruned on an ascendant simple cordon system. The vineyard soil is an acidic Cambic Umbrisol, with low levels of P and K, rich in N, low mineral colloids, and high fertility in the upper horizon. Grape berries and leaves were sampled on 4 consecutive days at green (E-L 34) and mature (E-L 38) stages,^29^ from 12 vines per replicate (4 replicates from 48 vines), placed in coolers, and transported to the laboratory. The exocarp of the berries was carefully separated from the mesocarp and used for plastid purification (Figure 1). From each vine, the seventh leaf from the apex was collected to ensure that measurements conducted on different dates correspond to leaves in a similar development stage.

### 2.2 Plastid Isolation and Chlorophyll Quantification

Plastids from grape berry skins and leaves at E-L 34 and E-L 38 were isolated as previously described.^5^ with minor modifications. Chlorophyll quantification was performed according to Lichtenthaler and Wellburn.^30^ Values were normalized by the total protein amount determined spectrophotometrically by the Bradford assay.^31^ Fluorescence microscopy images were acquired with a Leica Microsystems DM-5000B epifluorescence microscope.

### 2.3 Metabolomics Analysis

Metabolites were extracted using 100% (v/v) methanol, chloroform, and 50 mM Tris-HCl (1:2:1), spiked with 10 lg/mL dl-α-tocopherol acetate as the internal standard as reported by Frusciante et al. (2022).^32^ The extracts were analyzed with a Q-Exactive mass spectrometer (Thermo Fisher Scientific) coupled to an high-performance liquid chromatography (HPLC) system equipped with a photodiode array detector (Dionex). The chromatographic separation was performed as reported by Sulli et al. (2017)^33^ using liquid chromatography-mass spectrometry (LC-MS)-grade solvents (VWR international), while the atmospheric pressure chemical ionization (APCI) and high-resolution mass spectrometry (HRMS) parameters were set as previously described.^32^

For targeted metabolomics, metabolites were identified with online absorption spectra (except for lipids), by comigration with authentic standards (when available) and by accurate masses obtained from the Pubchem database (http://pubchem.ncbi.nlm.nih.gov/) for native compounds or from the Metabolomics Fiehn Lab Mass Spectrometry Adduct Calculator (http://fiehnlab.ucdavis.edu/staff/kind/Metabolomics/MS-Adduct-Calculator/) for adducts. The ion peak areas were normalized to the ion peak area of the internal standard (α-tocopherol acetate). Untargeted metabolomics was performed as previously described (Teixeira et al., 2020) using the SIEVE software (v2.2, Thermo Fisher Scientific, Waltham, MA).

### 2.4 Targeted Gene Expression

Total RNA was extracted from 200 mg of frozen ground grape berry skin and leaf samples, following the method described by Reid et al., 2006.^34^ For gene expression analysis by quantitative polymerase chain reaction (qPCR), 1 μg of total mRNA was converted to cDNA with an Xpert cDNA Synthesis Kit and oligo (dT) primers (Grisp Research Solutions). qPCR was performed in 96-well plates with Xpert Fast SYBR mastermix following the manufacturers’ instructions (Grisp Research Solutions). For each biological condition (*n* = 3), qPCR reactions were performed in triplicate (technical replicates). Gene expression was normalized to the transcript levels of the glyceraldehyde 3-phosphate dehydrogenase (*VvGAPDH*) reference gene.^35^ All primer sequences are detailed in Supplementary Table 1. The specificity of the qPCR reactions was checked through dissociation curves at the end of each qPCR reaction and data were analyzed using the CFX Manager Software (Bio-Rad Laboratories, Inc.).

### 2.5 Statistical Analysis and Bioinformatics

#### 2.5.1 Multivariate Analysis

Global nonpolar and polar compounds for untargeted metabolomics analyses were retrieved as previously described^36^ using the SIEVE software (v2.2, Thermo Fisher Scientific, Waltham, MA). To reduce the data dimensionality, an unsupervised Principal Component Analysis (PCA) and a supervised Partial least squares-discriminant analysis (PLS-DA) of leaf and berry skin plastids metabolomics data was performed in R software version 4.1.0 using the mixOmics package 6.16.3. The same procedure was used for both targeted isoprenoids and the lipid metabolomes of berry skin and leaf plastid samples. Heatmaps were performed with the ComplexHeatmap package (v 2.9.3) using the mean of four replicates. Bar plots of key genes involved in the biosynthesis of isoprenoids and lipids were performed with GraphPad Prism software, version 9.0. Statistically significant differences of each metabolite between mature and green samples of grape berries and leaves were determined using Student’s *t* test and marked with asterisks to denote the significance levels: *P ≤ 0.05; **P ≤ 0.01; ***P ≤ 0.001; and ****P ≤ 0.0001. Plot values were presented as mean values ± standard deviation (SD) of four biological replicates in each condition.

To display the main hubs of transcripts and metabolites, a network analysis was performed. Pearson correlations were calculated using MetScape plugin from Cytoscape version 3.8.2 (www.cytoscape.org) from a matrix of metabolite and transcript values of grape berries and leaves normalized on green stage values (Supplementary Table 2), as reported by Diretto et al. (2010)^37^ and Teixeira et al. (2020).^38^

The network diagram was generated with Pearson correlation values (ρ ≥ 0.90 and ρ ≤ −0.90) using a compound profuse force directed layout algorithm. Node strength (ns) calculation, intended as the average of all of the |ρs| yielded by each node, was used to identify the main elements (“hubs”) of the network (Supplementary Table 2). Positive and negative correlations were shown in red and blue, respectively. Different node shapes and colors were used to distinguish genes from metabolites. Finally, to better comprehend the metabolic changes of plastids, we used the MCODE plugin to obtain a series of metrical topological parameters of the network (density, cluster extrapolation, etc.), which may allow, on a mathematical basis, to infer biological information of transcript–metabolite cross-links.

## 3 Results

### 3.1 Untargeted Total Metabolome Changes in Plastids from Leaves and Berry Skins

Following the recently reported isolation and characterization of intact plastids from skins of green (E-L 34) and mature (E-L 38) grapes of the red cultivar “Vinhão”,^5^ in the present study, leaf tissues sampled at the same growth stages were also fractionated for metabolomics analysis of skin and leaf chloroplasts. Results from chlorophyll purification and quantification suggested that the fractionation protocols resulted in samples enriched in chloroplasts by up to 7-fold (Table 1). Chloroplasts isolated from leaves were preliminarily observed under the fluorescence microscope (Figure 1) to evaluate organelle integrity, as previously performed for chloroplast samples from berry skins.^5^ Subsequently, the metabolites present in the chloroplasts from skins and leaves at E-L 34 and E-L 38 were analyzed by LC-DAD-APCI-HRMS either at untargeted or targeted metabolomics levels.

**Table 1:** Chlorophyll (*a* + *b*) Content in Homogenates and Plastidial Fractions from Grapevine Leaves and Berry Skins^a^

	Chl (*a* + *b*) μg mg^–1^ prot.		
	homogenate	isolated plastids	fold change
leaves at E-L 34	92.9 ± 1.4^d^	482.1 ± 76.4^a^	4.9 ± 0.9
leaves at E-L 38	63.5 ± 8.0^cd^	360.6 ± 58.6^b^	5.7 ± 1.4
berry skins at E-L 34 (green)^b^	27.9 ± 1.7^cd^	199.1 ± 7.5^c^	7.3 ± 0.7
berry skins at E-L 38 (mature)^b^	–	223.7 ± 3.7^c^	–

a Superscript letters denote statistical significant differences tested by one-way ANOVA coupled to Tukey’s post hoc test.

b Data from ref (5).

First of all, an unsupervised principal component analysis (PCA) of untargeted data (Supplementary Table 2A) was performed to achieve a first evaluation of the samples: overall, PCA accounted for 77% of the total variation on the first two components and clearly separated green berry chloroplasts from the other samples (Supplementary Figure 1A). To maximize the covariance between the independent variables X (sample readings, the metabolomic data) and the corresponding dependent variable Y (samples), a supervised partial least squares-discriminant analysis (PLS-DA) was performed. PLS-DA showed a variation of 48% (PC1-*x* axis) in the plastid populations from green (E-L 34) *versus* mature (E-L 38) berries (Supplementary Figure 1B) and a variation of 44% (PC1-*x* axis) in chloroplast populations from E-L 34 *versus* E-L 38 leaves (Supplementary Figure 1C). Tentative identification of untargeted metabolomics revealed the presence of several lipids in the plastid samples, belonging to diacylglycerols, fatty acids, or sterol derivatives (Supplementary Table 2B).

### 3.2 Changes in Plastid Isoprenoids in Berry Skins at Green and Mature Stages

**Figure 2:**
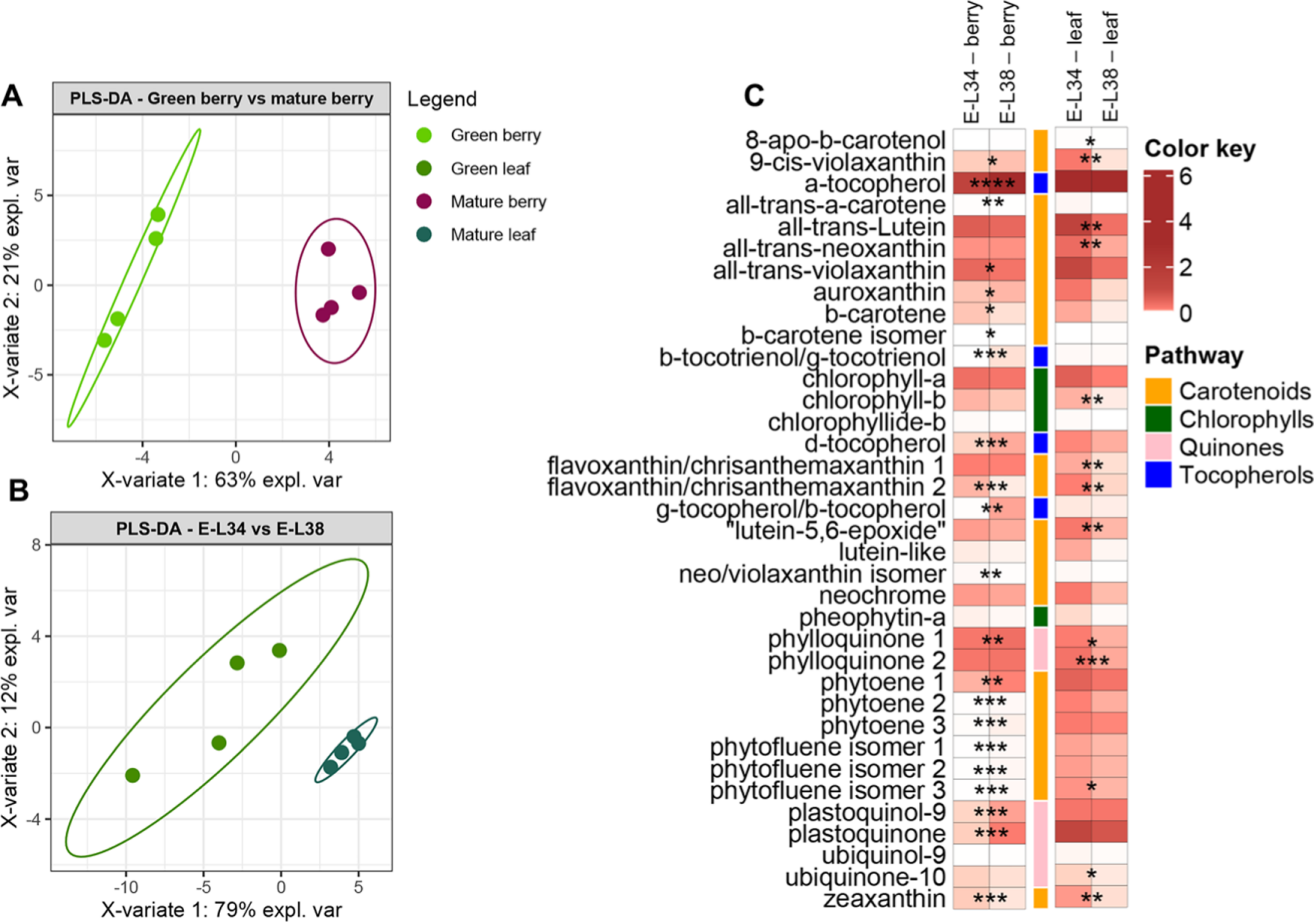
Changes in targeted isoprenoids from purified plastids from green leaf and berry skin of red grapes cv. “Vinhão”: supervised partial least squares-discriminant analysis (PLS-DA) of (A) green berry *vs* mature berry, (B) green leaf *vs* mature leaf, and (C) heatmap of the observed changes. Variables in the PLS-DA score plot were colored according to the tissue and developmental stage. Data represent, for each metabolite, the fold on the internal standard (IS, a-tocopherol acetate) level/gm DW. Asterisks indicate statistical significance between mature (E-L 38) and green (E-L 34) conditions following the Student’s *t*-test: *P ≤ 0.05; **P ≤ 0.01; ***P ≤ 0.001; and ****P ≤ 0.0001.

Since isoprenoids are the typical secondary compounds accumulating in the plastids, a targeted metabolomics analysis was performed to identify and quantify the different isoprenoid classes in berry skin samples. The obtained dataset, for a total of 37 compounds, included chlorophylls: the precursor chlorophyllide *b* and the chlorophyll catabolite pheophytin *a*; tocochromanols: α- and β-/γ-tocopherol; quinones: phylloquinone, ubiquinol-9, plastoquinol-9, ubiquinone-10, and plastoquinone; and carotenoids: β-carotene, and ϵ−β- and β–β-xanthophylls (Supplementary Table 3). An unsupervised PCA score plot of the first two components explained 84% variation and immediately discriminated the metabolomic profiles of the two populations of isolated plastids from leaves (E-L 34 *versus* E-L 38 stages), whereas the distinction separation from skin plastids was less evident because of the large scale of PC1 (*x* axis) (Supplementary Figure 2A). On the contrary, the supervised PLS-DA analysis allowed for a clear discrimination of the two populations of isolated plastids from skins with a variation of 63% (PC1-*x* axis) in E-L 34 *versus* E-L 38 samples (Figure 2A), and a variation of 79% (PC1-*x* axis) in isolated plastids from E-L 34 *versus* E-L 38 green leaves (Figure 2B).

PLS-DA loadings showed that 13 of the top 15 compounds were overrepresented in plastids from berries at E-L 38 compared to E-L 34. That is, β-tocotrienol/γ-tocotrienol and β-tocopherol/γ-tocopherol increased 103- and 67-fold respectively, plastoquinone 7-fold, and phytoene and phytofluene isomers up to 4- and 10-fold, respectively. In our experimental conditions, the two most accumulated metabolites in the plastids of berry skins at E-L 34 were flavoxanthin and zeaxanthin, at 0.9- and 0.5-fold, respectively (Figure 2C, Supplementary Figure 3A). Chlorophylls *a* and *b*, and related degradation products, showed no significant differences between mature (E-L 38) and green (E-L 34) stages in purified plastids from berry skins (Figure 2C), supporting previous observations (Table 1 and Figure 1; Teixeira et al. 2022)^5^ that there is no loss of chloroplasts during the transition in color of the fruit after veraison.

While in grape skins, some isoprenoids were more accumulated in chloroplasts at the mature stage than at the green stage, in leaves, all compounds (24) that significantly changed their levels in plastids were overrepresented at the E-L 34 stage (Figure 2C, Supplementary Figure 3B). For instance, the total amount of phytoene isomers, a key precursor of various carotenoids, was 2-fold higher in isolated plastids from leaves at E-L 34 than at E-L 38, while in berry skin plastids, the same compound accumulated 2.3-fold during the transition from the green to the mature stage. Similarly, the subsequent compound in the carotenoid pathway, phytofluene isomer 1, was 10-fold higher in mature berry skin plastids, contrary to plastids from leaves at the same stages. However, nearly half of the identified isoprenoids in plastids from either skins or leaves did not change between E-L 34 and E-L 38, including chlorophyll *a*, chlorophyll *b*, pheophytin *a*, all-*trans*-violaxanthin, and a neo/violaxanthin isomer (Figure 2C).

**Figure 3:**
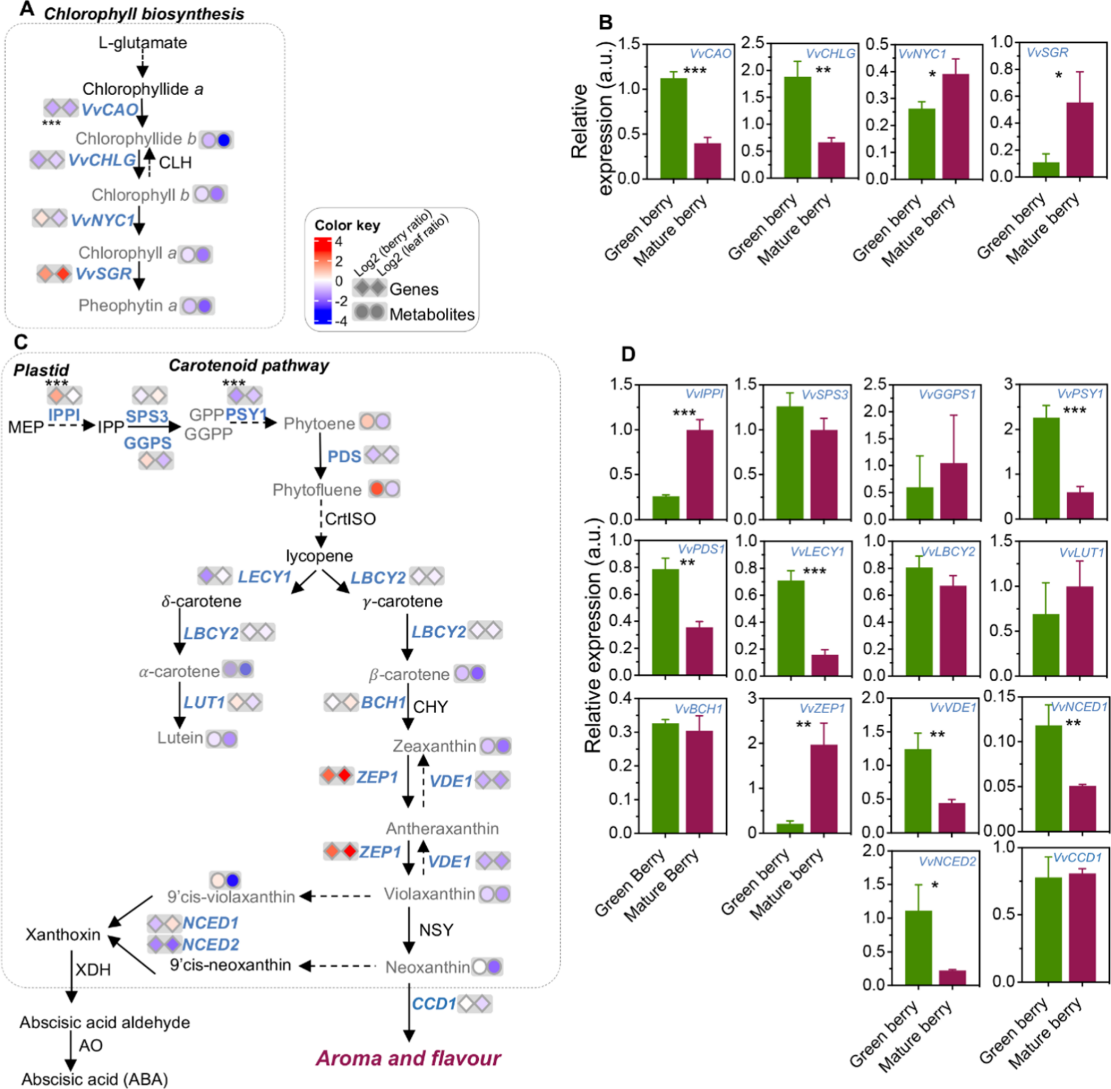
Relative content of isoprenoids from purified plastids and transcripts expression of key genes of green and mature berry skin of red grape berry cv. “Vinhão”, involved in the biosynthesis of isoprenoids. (A) Chlorophyll biosynthesis pathway. (B) Relative expression of key genes involved in the biosynthesis of chlorophylls. (C) Isoprenoid biosynthesis pathway with the ratio of metabolites (log2 green/mature) identified in purified plastids from the skin of grape berries and leaves at green (E-L 34) and mature (E-L 38) stages of development. (D) Relative expression of key genes involved in isoprenoid biosynthesis. Gray metabolite and blue gene names indicate measurements from the present study. Asterisks indicate statistical significance between mature (E-L 38) and green (E-L 34) stages following the Student’s *t*-test: *P ≤ 0.05; **P ≤ 0.01; and ***P ≤ 0.001.

To better understand the developmental changes of the different isoprenoid classes in the two tissues under study, transcript levels of key genes of each pathway were measured by qPCR, and figures combining gene expression and metabolic results were depicted, by comparing the mature *vs* green stage for each tissue, and expressed as log2 data. Genes involved in the biosynthesis and degradation of chlorophylls in grape berry skin are shown in Figure 3A,B. The expression of *VvCAO* and *VvCHLG*, encoding *chlorophyllide-a oxygenase/chlorophyll b synthase* (CAO) and *chlorophyll synthase* (CHLG), was downregulated by 65% at E-L 38 (Figure 3B). Coincidentally, the expression of *VvNYC*1 and *VvSGR*, encoding *chlorophyll(ide) b reductase* (NYC) and *magnesium dechelatase* (NYC-STAY-GREEN), was upregulated in berries by 49 and 403%, respectively (Figure 3B). In leaves, *VvNYC*1 expression did not change from E-L 34 to E-L 38, whereas *VvSGR* was characterized by a 1400% increase (Supplementary Figure 5A).

The transcriptional profiles of carotenoid biosynthesis genes in the berry skins are depicted in Figure 3C,D. The expression of *VvIPPI*, encoding *isopentenyl-diphosphate delta-isomerase*, was upregulated by 286% in berry skins at mature stage (E-L 38), contrary to *VvPSY1* and *VvPDS1*, encoding a *phytoene synthase* and a *phytoene desaturase* (PDS) that were downregulated by 73 and 55% respectively. *VvSPS1* (*Solanesyl diphosphate synthase 1*) and *VvGGPS1* (*Geranylgeranyl diphosphate synthase*) transcripts, encoding intermediate biosynthetic steps of the key metabolite phytoene, showed similar abundance in the skins of green (E-L 34) and mature (E-L 38) grape berries (Figure 3C,D). A similar tendency was observed for most of the late genes in the pathway (*lycopene* β*-cyclase 2*, *VvLBCY2; Carotene* ε*-hydroxylase (cytochrome P450)*, *VvLUT1*; and β*-carotene hydroxylase 1*, *VvBCH1*). Notable exceptions were represented by the transcript levels of *VvLECY1*, encoding *lycopene* ε*-cyclase*, involved in the biosynthesis of δ-carotene, decreased by 76% in the transition from E-L 34 to E-L 38; and by the expression of *VvVDE1*, encoding a *violaxanthin deepoxidase*, which decreased by 64% in the transition from E-L 34 to E-L 38. In turn, *VvZEP1*, encoding a *zeaxanthin epoxidase*, was upregulated by 855%, suggesting a fine-tuned regulation of the xanthophyll cycle (Figure 3C,D). To explore similarities between carotenoid synthesis in berry skins and leaves, the expression pattern of the above-referred genes was also evaluated in leaves at E-L 34 and E-L 38 (Supplementary Figure 5). Of note, several genes, including *VvPSY1*, *VvZEP1*, and *VvVDE1* showed a similar expression pattern in berry skins and leaves in the transition from E-L 34 to E-L 38.

At the carotenoid catabolism level, the expression of *VvNCED1* and *VvNCED2*, encoding *9-cis-epoxycarotenoid dioxygenases* involved in the biosynthesis of the ABA precursor xanthoxin, was downregulated by 57 and 80% during the transition of berries from E-L 34 to E-L 38, while *VvCCD1*, encoding a *carotenoid cleavage dioxygenase 1*, involved in the biosynthesis of the aromatic apocarotenoids as β-ionone, did not change.

Quinone biosynthesis in the berry skin also changed from the green to the mature stage because the expression of *VvHGGT*, *VvGGR*, and *VvVTE3* genes was downregulated during the transition from E-L 34 to E-L 38, while *VvVTE4* was upregulated (Supplementary Figure 4A,B). When the expression of these genes was studied in leaf samples, results showed a similar transcriptional reprogramming (Supplementary Figure 5B).

### 3.3 Lipid Biosynthetic Changes in Plastids at Two Developmental Stages

**Figure 4:**
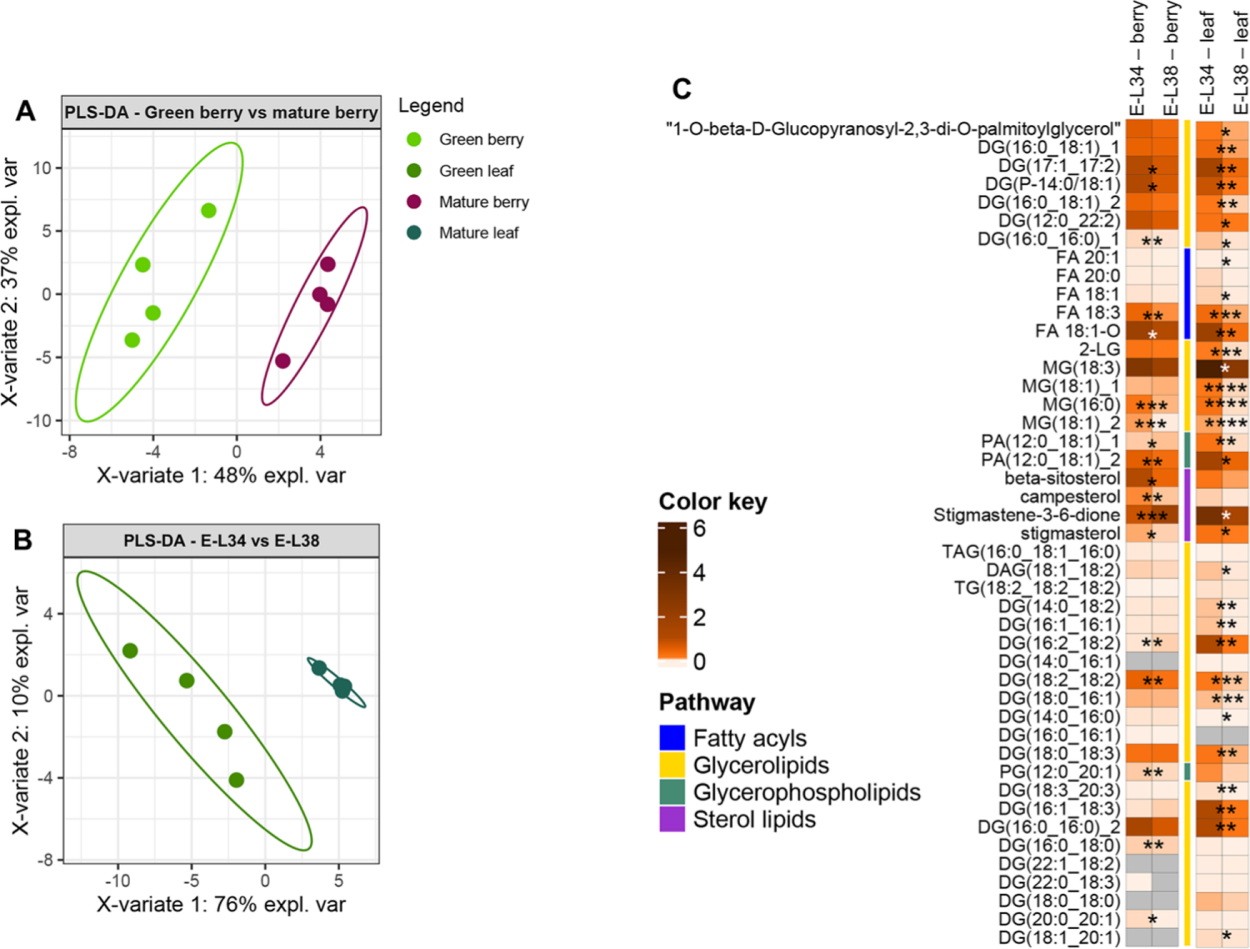
Modifications observed in targeted lipids present in plastids purified from green leaf and berry skin of red grapes cv. “Vinhão”: Supervised Partial Least Squares-Discriminant Analysis (PLS-DA) of (A) green berry *vs* mature berry, (B) green leaf *vs* mature leaf, and (C) heatmap of the observed modifications. Variables in PLS-DA score plot were colored according to the tissue and developmental stage. Asterisks indicate statistical significance between mature (E-L 38) and green (E-L 34) conditions following the Student’s *t*-test: *P ≤ 0.05; **P ≤ 0.01; ***P ≤ 0.001; and ****P ≤ 0.0001.

Targeted metabolomic analysis identified 45 lipids involved in plastid and, more in general, in cell metabolism belonging to the following categories: fatty acyls (FA), sterol lipids (ST), glycerophospholipids (GP), and glycerolipids (GL) [monoacylglycerols (MAGs), diacylglycerols (DAG), and triacylglycerols (TAG)]. The unsupervised PCA score plot of the first two components explained 70% of variation, discriminating the lipid metabolic profiles of plastids from leaves at E-L 34 and E-L 38, albeit the separation of the two populations of plastids from berry skins was less evident (Supplementary Figure 2B). However, the supervised PLS-DA analysis clearly discriminated the lipid profiles of the two plastid populations from berry skins with a variation of 56% (PC1-*x* axis) in isolated plastids from E-L 38 *versus* E-L 34 stages (Figure 4A). The lipid profiles of the two plastid populations from leaves were also clearly discriminated, with a variation of 75% (PC1-*x* axis) in isolated plastids from E-L 38 *versus* E-L 34 stages (Figure 4B). Most of the identified lipids in plastids from berry skins that showed significant changes between green and mature stages were impoverished at the E-L 38 (mature stage) (e.g., glyceryl monostearate [MG(18:1)] and palmitic-glycerol [MG(16:0)] decreased by 96 and 77% respectively), while only stigmastene-3,6-dione (119%) and oleolyl-glycerol [MG(18:1)] (22%) were more represented at the mature stage (Figures 4C and S3C). Notably, all lipids identified in plastids isolated from leaves decreased during the transition from E-L 34 to E-L 38 (Supplementary Figure 3D).

**Figure 5:**
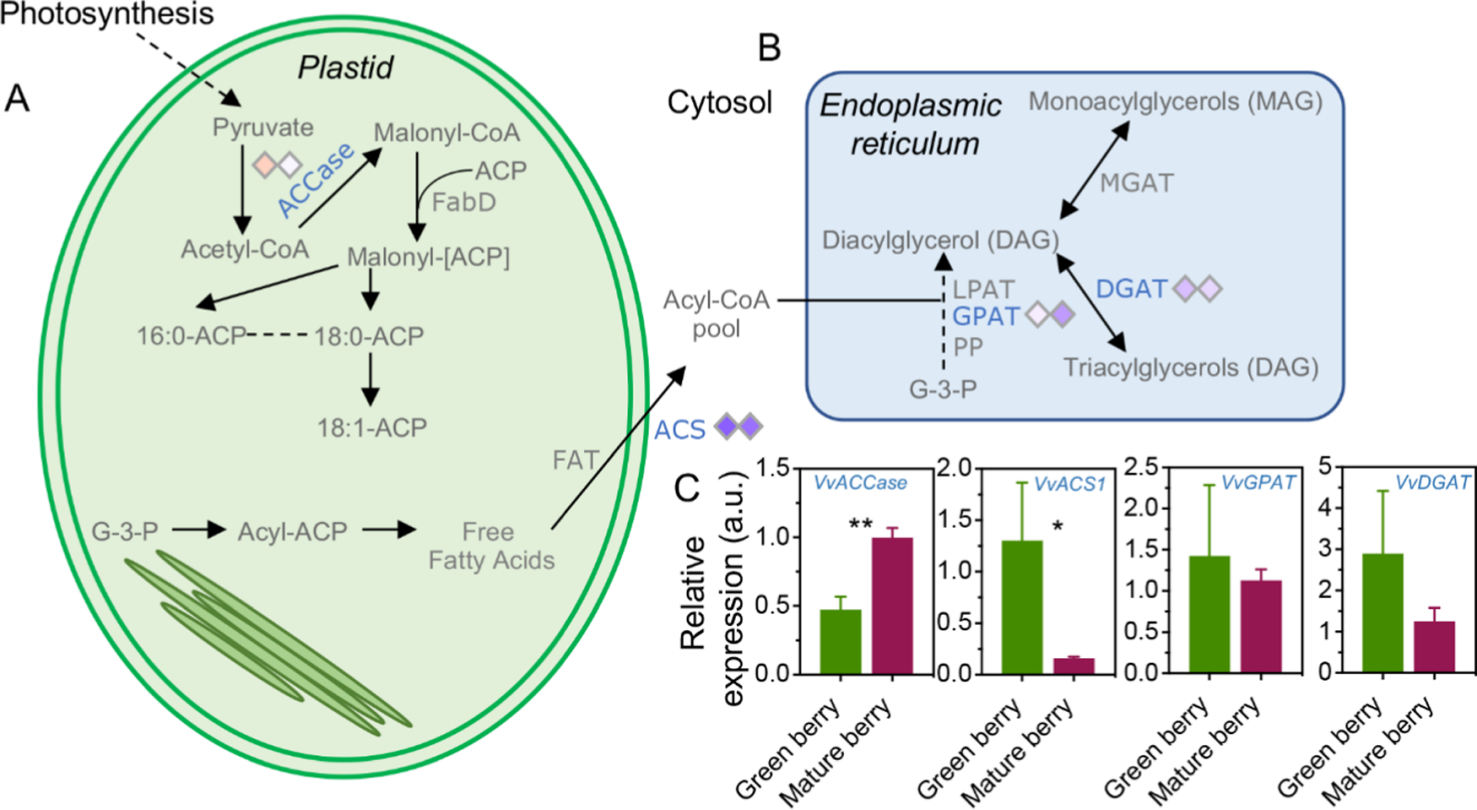
Lipids and targeted key genes from grape berries and leaves. (A) Simplified schematic of lipid biosynthesis in plastid and (B) endoplasmic reticulum, and (C) expression of key genes involved in lipid biosynthesis. Asterisks indicate statistical significance between mature (E-L 38) and green (E-L 34) conditions following the Student’s *t*-test: **P ≤ 0.01.

In terms of gene expression alterations, transcript levels of *VvACCase*, encoding an *acetyl-CoA carboxylase* (*ACCase*), increased by 111% in berry skins during the transition from E-L 34 to E-L 38, while the expression of *ACS1*, encoding an *acetyl-CoA synthetase* (*ACS1*), was downregulated by 88%. On the contrary, the expression of *VvGPAT* and *VvDGAT*, encoding a *glycerol-3-phosphate acyltransferase* (*GPAT*) and a *diacylglycerol acyltransferase* (*DGAT*) involved in the biosynthesis of DAG and TAG lipids, showed no significant differences in berry skins at E-L 34 and E-L 38 (Figure 5A,B).

Finally, genes encoding key enzymes of lipid metabolism in leaves showed a general downregulation during the transition from E-L 34 to E-L 38. Among those, *VvACS1* and *VvGPAT* expression showed the most significant reduction (by 84 and 73%, respectively) (Figure 5C).

### 3.4 Transcript–Metabolite Correlation is Positive in Mature Grape Berries and Leaves

**Figure 6:**
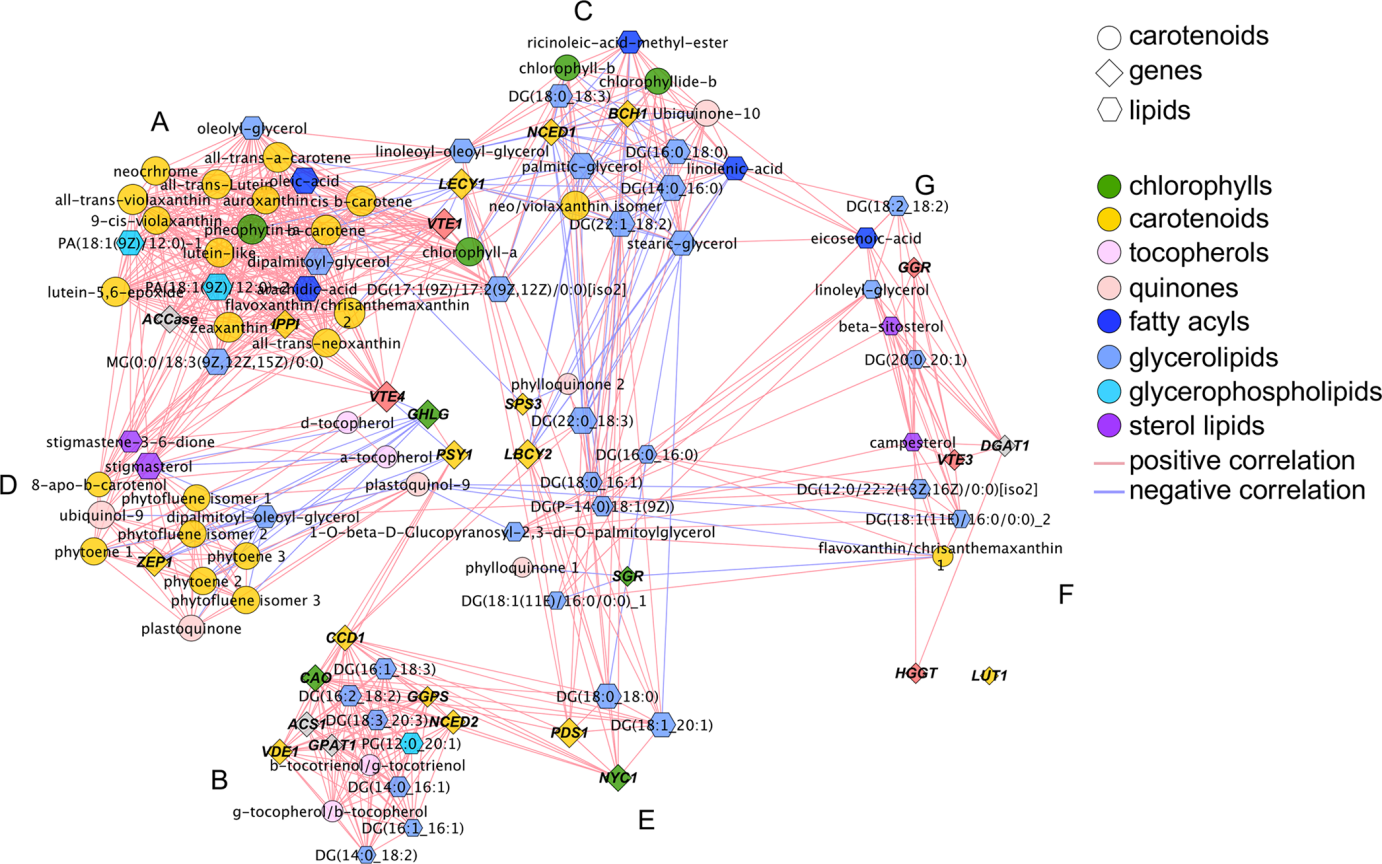
Correlation network of transcripts and metabolites from plastids purified from grapevine berry skins of red grapes and leaves of cv. “Vinhão” at mature stages of grape berry development normalized to green stages. Network is visualized with lines joining the nodes (edges) representing correlations: direct (positive) correlations are shown in red, while inverse (negative) correlations are shown in blue. Edge thickness is proportional to the respective correlation value (ρ). Node shapes represent a gene transcript (diamonds) or a metabolite (circles—isoprenoids; hexagons—lipids) from different biosynthetic pathways. For more details, see Materials and Methods and Supplementary Table 5.

The relationships between transcripts and metabolites in berries skins and leaves at E-L 38 (mature stage) were explored through a correlation analysis using a matrix (Supplementary Table 5) and a correlation network (Figure 6) of Pearson correlation coefficient values (ρ). The overall network strength was moderate to strong (|ρ| = 0.55), (Figure 6, Table S4). In the network diagram, the MCODE Cytoscape plugin enabled the identification of seven subclusters (Figure 6, Supplementary Figure 6), with the largest one showing only positive correlations, and with notable members as *VvIPPI*, *VvVTE1*, and *VvACCase*, which positively correlated to 21 metabolites belonging to isoprenoids (chlorophylls and carotenoids) and lipids (triacylglycerols, monoacylglycerols, and diacylglycerophosphates) (Figure 6, Supplementary Figure 6A). Similarly, the second largest subcluster showed only positive correlations, where *VvGGPS1*, *VvCAO*, *VvVDE1*, *VvNCED2*, *VvCCD1*, and *VvACS1* were correlated with six lipids (five glycerolipids; one glycerophospholipid) and two tocopherols (Figure 6, Supplementary Figure 6B). Notably, *VvCLHG*, *VvVTE4*, and *VvPSY1* connected the second and first clusters.

The third largest cluster included *VvLECY1*, *VvBCH1*, and *VvNCED1*, involved in the biosynthesis of carotenoids, apocarotenoids, and lipids, which showed only negative correlations with isoprenoids (chlorophyllide-*b*, neo/violaxanthin isomer, and ubiquinone-10) and two categories of lipids: fatty acyls (ricinoleic-acid-methyl-ester [FA 18:1;O] and linolenic acid [FA 18:3]) and seven glycerolipids. Glycerolipids of particular note were palmitic-glycerol [MG(16:0)] and glyceryl monostearate [MG(18:1)], which positively correlated between each other (ρ = 0.98) (Figure 6, Supplementary Figure 6C). Similar to the previous clusters, *VvVTE1* and *VvLECY1* bridged the first and third clusters together with chlorophyll *a* and two glycerolipids.

Except one glycerolipid (sharing exclusively negative correlations), only positive correlations were observed in the case of the fourth cluster, where *VvZEP1* correlated with isoprenoids and two quinones (Figure 6, Supplementary Figure 6D). In the fifth cluster, only positive correlations were observed, with *VvNYC1* and *VvPDS1* correlated with two glycerolipids (Figure 6, Supplementary Figure 6E). Similarly, *VvDGAT*, involved in the biosynthesis of triacylglycerols, correlated with campesterol (ρ = 0.97), while *VvVTE3* positively correlated with two glycerolipids (Figure 6, Supplementary Figure 6F). Finally, a positive correlation was also observed in the seventh cluster between *VvGGR* and a sterol lipid (up to ρ = 0.94) and a glycerolipid (up to ρ = 0.98) (Supplementary Figure 6E), suggesting a close relationship between the biosynthesis of antioxidants and lipids.

## 4 Discussion

Our proteomic analysis of isolated chloroplasts of berry skins of cv. “Vinhão” suggested that chloroplasts are more involved in biosynthetic reactions in green berries (accumulating proteins involved in the Calvin cycle) than in mature berries, when proteins of the light reactions significantly accumulate. In the present study, the results confirmed that plastids from the grape skins are pivotal in the synthesis of important berry compounds like isoprenoids, including chlorophylls, and lipids. This biosynthetic activity may serve to preserve the photosynthetic machinery of grape skin chloroplasts during development and ripening (Table 1, Figure 1, Teixeira et al. 2022).^5^ Because Calvin cycle proteins were found to be impoverished in skin plastids at the mature stage (Teixeira et al. 2022),^5^ cytosolic precursors like isopentenyl pyrophosphate (IPP) may feed the synthesis of some isoprenoids, including phytoene or phytofluene, that accumulate at the mature stage, as shown in the present study.

Metabolomic and transcriptomic data suggested that berry and leaf chloroplasts share conserved mechanisms of synthesis and degradation of chlorophylls, at least from E-L 34 (green stage) to E-L 38 (mature stage). However, degradation of chlorophylls likely starts earlier in leaves than in grapes because from E-L 34 to E-L 38, the amounts of both chlorophyll *a* and *b* decreased in leaves but not in grape berry skins (Table 1, Figure 2). In parallel, the steady-state transcript levels of *VvCAO* and *VvCHLG*, involved in key steps of chlorophyll biosynthesis, were downregulated in leaves (as well as in berry skins) during the transition from E-L 34 to E-L 38, suggesting that *de novo* chlorophyll biosynthesis decreases toward E-L 38. Concordantly, the expression pattern of the *SGR* gene increased from E-L 34 to E-L 38. This gene encodes for a STAY-GREEN 1 protein required for the dismantling of photosynthetic *chl*–protein complexes^39^ and the initiation of chlorophyll *a* breakdown into pheophytin *a* and *b*.^40,41^ Apart from these similarities between chlorophyll biosynthesis and breakdown in leaves and berries, the esterification step of chlorophyllide-*a* and *b* with phytyl or geranylgeranyl pyrophosphate seems active in the skins at E-L 38, while the transcription of *VvIPPI* gene is repressed in leaves at the same stage. Contrasting results were also observed when the levels of pheophytin *a*, the first product of chlorophyll demetallation, were found significantly higher in plastids from leaves at E-L 34 than at E-L 38, while no differences were observed between plastids from skins of green and mature berries. This suggests that senescence begins earlier in leaves than in berries.

A peak of carotenoids was observed 2 weeks before veraison in Chardonnay and, at veraison, in Cabernet Sauvignon, when the maximum expression of the β*-carotene hydroxylase* coding gene occurred.^42^ Two unigenes encoding *phytoene synthase* (*PSY*) were downregulated in Cabernet Sauvignon whole berries during the transition from the green to the mature stage^43^ in accordance with other studies.^44^ Similarly, our results also showed that the expression of *VvPSY* and *VvPDS* was higher in the skin of the berry at E-L 34 than at E-L 38, a finding that might explain the overaccumulation of the key metabolite phytoene (and of two of its isomers) in plastids from the skins at the mature stage. Contrarily, late compounds of the carotenoid pathway (e.g., β-carotene, zeaxanthin, all-trans-violaxanthin) were found to be more accumulated in grape skin plastids at E-L 34 than at the mature stage, which correlated with the expression patterns of *VvLECY1*, *VvLBCY2*, and *VvVDE1* genes. Carotenoids like β-carotene, violaxanthin, antheraxanthin, zeaxanthin, and lutein are mainly present in the reaction center of photosystem II and in the antenna systems of light-harvesting complexes,^45^ thus stabilizing and protecting the photosynthetic apparatus during high-light stress.^46^

The xanthophyll cycle of higher plants is an important photoprotection mechanism during periods of high-light illumination.^47,48^ In this context, the higher expression of *VvZEP1* in the skin of grape berry at E-L 38 indicates that, in mature grapes, the epoxidation reaction mediated by the zeaxanthin epoxidase (ZEP) is favored over the enzymatic deepoxidation of violaxanthin to zeaxanthin, which corresponds with the observed higher amounts of 9-*cis*-violaxanthin measured in grape skin plastids at E-L 38. Thus, these findings provide clues that mechanisms of protection of the photosynthetic machinery are still important at the mature stage. Also supporting this hypothesis, quinones and tocopherols accumulated at higher levels in the plastids of the skins at the mature stage, which correlated with the observed upregulation of *VvVTE1* and *VvVTE4* during the transition from E-L 34 to E-L 38. Indeed, several studies demonstrated that tocopherols, in cooperation with the xanthophyll cycle, act to preserve PSII from photoinactivation and protect membrane lipids from photooxidation.^49,50^ In turn, ubiquinone and plastoquinone are fundamental electron carriers in oxidative phosphorylation and photosynthesis, respectively.^50^

Carotenoids play a pivotal role as precursors of abscisic acid (ABA) in higher plants. Indeed, ABA is produced from the cleavage products of 9-*cis*-violaxanthin and 9-*cis*-neoxanthin.^51^ Our results showed isomers of violaxanthin (9-*cis*-violaxanthin precursors) were more abundant in plastids from the skins of berries at E-L 34, which aligns with the observed upregulation of *VvNCED1* and *VvNCED2* genes. Conversely, 9-*cis*-violaxanthin was found at higher levels in plastids from skins at E-L 38. It has been described that the rate-limiting step for ABA biosynthesis in leaves is the cleavage of 9-*cis*-epoxyxanthophylls by the NCED dioxygenase,^51−53^ and a similar phenomenon might also occur in grape berry skins. Moreover, it appears that endogenous ABA decreases progressively in the flesh, while it accumulates in the skin from the beginning of the color change to maturity,^54^ strongly contributing to berry maturation. This finding matches with the aforementioned higher levels of 9-*cis*-violaxanthin in plastids from mature berries.

Grape aromas result from intermediates of glycolysis via isoprenoid/carotenoid metabolism and via acetyl-CoA/fatty acid metabolism.^42^ The carotenoid cleavage dioxygenase (CCD) family has been shown to produce important volatile flavor and aroma apocarotenoids including β-ionone, geranylacetone, pseudoionone, α-ionone, and 3-hydroxy-β-ionone in a range of plant species.^55^ In Cabernet Sauvignon grapes, the transcript abundance of genes involved in carotenoid or fatty acid metabolism, including *VvCCD1*, increased at late maturation stages (at higher °Brix levels) and was higher in berry skins than in flesh.^56^ In the present study, a similar abundance of *VvCCD1* was found in the skins of berries at E-L 34 and E-L 38, while, on the contrary, *VvCCD1* expression in leaves decreased from E-L 34 to E-L 38. However, it was shown that the overexpression or silencing of *VvCCD1* in transgenic grapevines did not influence leaf norisoprenoid levels,^55^ thus suggesting the involvement of additional CCD members (from CCD1 or CCD4 families).^57^

Other important grape flavors are the ones derived from the fatty acid metabolism pathway, which leads to the production of aromatic alcohols (e.g., hexenol and benzyl alcohol) and esters.^56^ Palmitic, myristic, and lauric acids were reported to be the most abundant lipids in 12 commercial red wines made from grapes differing in variety and vintage.^58^ However, most of the published studies regarding grapevine lipids are based on lipidomic profiles focused on the elucidation of biotic or abiotic stress effects on grapevine leaves rather than on berry samples (reviewed in refs (59) and (60)). An exception is represented by an older study that showed that the most abundant fatty acid residues in grape flesh and skins are linoleic acid, palmitic acid, and linolenic acid.^61^ Another exception is more recent, research on seven grapevine cultivars, including the red variety Tempranillo, that identified several free fatty acids (e.g., palmitic, stearic, linolenic, or arachidic acids), glycerolipids (e.g., 1-oleoyl-*rac*-glycerol), and triterpenoids (e.g., oleanolic acid)^62^ in grape berries. Small changes in fatty acid profiles were observed at different stages of berry development, with the exception of neutral and glycolipids containing linolenic acid residues, which decreased.^63^ In the present study, several of the abovementioned lipids present in the four categories were found in isolated plastids from berry skins of red grapes, which were much more abundant at green than at mature stage, and a similar profile was observed in leaves. Although some of the lipids identified in this study are not typically synthesized or accumulated in the plastid, and thus may be due to sample contamination, a large amount of plastid-specific glycerolipids were also identified.

Some of the sterol lipids were previously identified in the wax matrix of grape berries (e.g., sitosterol, stigmasterol, campesterol) in red (cv. “Vinhão”) and white (cv. “Loureiro”). The relative amounts of specific compounds, mainly stigmasterol, tremulone, and oleanolic acid, correlated well with the composition of the microbial communities of the berry surface.^28^ The polyunsaturated α-linolenic acid (fatty acyls) is a substrate for lipoxygenase (LOX) and hydroperoxide lyase activities, which forms C6-aldehydes and alcohols, responsible for the “green flavor”.^64^ Consistently, its abundance decreased during the transition from the green to the mature stage in the present study.

Plastid-specific glycerolipids are important in the biosynthesis of chloroplasts by contributing to the formation of the stacked thylakoid membranes.^65,66^ In accordance, our results showed that their relative abundance in the chloroplasts of berry skins decreased during the transition from the green to the mature stage when the *de novo* synthesis of plastids is likely decelerating, as previously suggested. In this context, a study on cv. ‘Ribolla Gialla’ berries also showed that several subclasses of glycerolipids (MG, DGDG, and MGDG) are less abundant in the berry at the mature stage, at high °Brix values.^67^

The photosynthesis and accumulation of isoprenoids are sensitive to environmental conditions. In a previous study, photosynthesis and isoprene accumulation responded negatively to elevated O_3_ (−8 and −10%, respectively) and drought (−15 and −42%) and in opposite ways to elevated CO_2_ (−23 and +55%) and warming (+53 and −23%, respectively).^68^ Thus, environmental factors like temperature or water availability may have influenced gene expression and metabolite accumulation in the present study. Other fluctuations in metabolites and negative correlations between transcripts and metabolites observed in the present study may also have resulted from the complexity of the mechanisms of gene expression and regulation. For instance, an enzyme with a specific biological function may be coded by a family of genes that can show different expression patterns during grape berry development and maturation and in response to environmental conditions. Furthermore, a specific biological function may be inhibited at the protein level when the corresponding gene(s) is (are) highly upregulated, which leads to a mismatch between gene expression and the amount of metabolites.

The network strength (|ρ| = 0.55) and the high number of positive correlations observed between genes and metabolites suggests a well-coordinated reprogramming of the different isoprenoid classes at transcript–transcript, metabolite–metabolite, and transcript–metabolite levels from the green to the mature stage. In fact, the strong correlations between metabolite and transcript levels more likely reflect metabolite regulation of transcription than vice versa.^69^*VvCHLG*, *VvVTE1*, *VvPSY1*, *VvLECY1*, and *VvLBCY2* transcripts occupied core positions connecting A, B, C, and D clusters, suggesting that these genes display a preponderant influence in the regulation of isoprenoid biosynthetic routes. PSY is a key enzyme of the carotenoid biosynthetic pathway, catalyzing the head-to-head condensation of two molecules of GGPP leading to the production of phytoene, the precursor for all other carotenoids in nature.^70^ Previous studies showed that *PSY1* genes modulate carotenoid biosynthesis in tomato.^43,44^ The strong transcript–transcript positive correlations observed in seven genes (subcluster D) involved in the biosynthesis of chlorophyll (*VvCAO*, *VvCHLG*), carotenoids precursors (*VvGGPS1*), ABA precursors (*VvNCED2*), aromas precursors (*VvCCD1*), and lipids (*VvACS1*, *VvGPAT1*) suggest that the biosynthetic metabolic steps of the different classes of isoprenoids and lipids may share common regulatory mechanisms in plastids and leaves, as many transcripts clustered closely together in the same subcluster. The results of a series of previous studies have found a strong connection between plastidial isoprenoids and lipids. For instance, it has been shown that chromoplast carotenoid composition greatly affects lipid qualitative and quantitative occurrence, which are needed to better enable carotenoid sequestration.^71^ Other studies have proven that LOX enzymes can use both lipids and carotenoids as substrates, thus confirming a close relationship between the two classes of compounds, affecting wheat grain color^72^ and apocarotenoid synthesis and accumulation.^73^

The significance of the present study lies both in the relevance of the scientific question and in the chosen experimental approach of performing metabolomics and gene expression in plastids isolated from berry skins and leaves (comparative approach), and integrating all data through correlation analyses between metabolites and transcripts. Our results showed that plastids that persist at maturity in the grape berry skins and leaves share conserved mechanisms of synthesis (and degradation) of important components of the photosynthetic machinery, some of which have important roles in grape and wine sensory characteristics. These components include chlorophyll pigments, their precursors and catabolites, carotenoids, quinones, and lipids. Thus, the berry is not merely a typical sink. The observed positive correlations between key transcripts of isoprenoid and lipid biosynthesis and metabolites of those pathways pave the way for future studies aimed to identify key metabolic markers for breeding-specific metabolic traits. These approaches and results also encourage studies in other fleshy fruits, like tomato, that accumulate lycopene at the mature stage.

## Supporting Information Available

Unsupervised and supervised analysis of untargeted metabolomic data (Figure S1); principal component analyses of targeted metabolomic data (Figure S2); plot loadings of PLS-DA analysis (Figure S3); metabolites and key transcripts of quinone biosynthesis (Figure S4); relative expression of key genes of grapevine leaves (Figure S5), and subnetworks generated using the MCODE Cytoscape plugin applied to the global correlation network shown in Figure 7 (Figure S6) (PDF)
List of primers (Table S1); list of differentially accumulated metabolites (DAMs) identified in purified plastids from red grape berry skins and leaves at green (E-L 34) and mature (E-L 38) developmental stages (Table S2A); sublist of differentially accumulated metabolites (DAMs) subjected to the identification pipeline (Table S2B); mean of targeted isoprenoids identified in purified plastids from red grape berry skins and leaves at green (E-L 34) and mature (E-L 38) developmental stages (Table S3); mean expression of transcripts from the berry skins and leaves at green (E-L 34) and mature (E-L 38) developmental stages (Table S4); and Pearson correlation matrix of targeted isoprenoids, lipids, and targeted gene transcripts identified in purified plastids from red grape berry skins and leaves at the green stage (E-L 34) (Table S5) (XLSX)
