# Supplementary material for: Biosynthesis of Chlorophyll and Other Isoprenoids in the Plastid of Red Grape Berry Skins
Source: J Agric Food Chem. 2023 Jan 18;71(4):1873–85. doi: 10.1021/acs.jafc.2c07207 (PMC9896546; doi:10.1021/acs.jafc.2c07207)
Supplement: Supplementary file 1 — jf2c07207_si_001.pdf [file jf2c07207_si_001.pdf]

**Supporting Information**

**Biosynthesis of chlorophyll and other isoprenoids in the plastid of red grape berry skins**

António Teixeira[1]*, Henrique Noronha[1]*, Sarah Frusciante[2], Gianfranco Diretto[2] and Hernâni Gerós[1]

[1] Centre of Molecular and Environmental Biology, Department of Biology, University of Minho, Braga, Portugal, [2]Casaccia Research Center, ENEA, Italian National Agency for New Technologies, Energy.

*Corresponding authors: antonio.teixeira@bio.uminho.pt; henriquenoronha@bio.uminho.pt

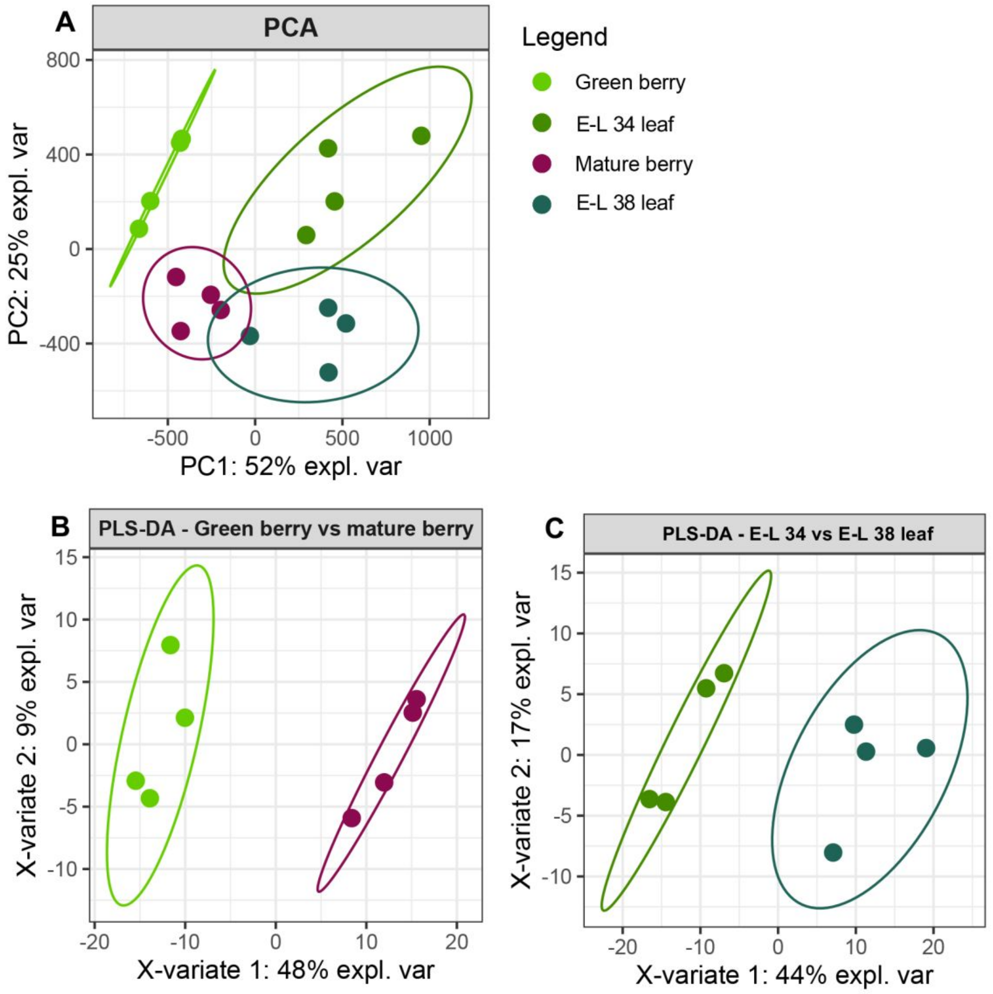


**Supplementary Figure 1.** Untargeted metabolomic data from leaf and berry exocarp plastids purified from cv. “Vinhão”. (**A**) Unsupervised Principal Component Analysis (PCA) and **B, and C**) supervised Partial Least Squares-Discriminant Analysis (PLS-DA). Variables in score plot were colored according to the tissue and developmental stage.

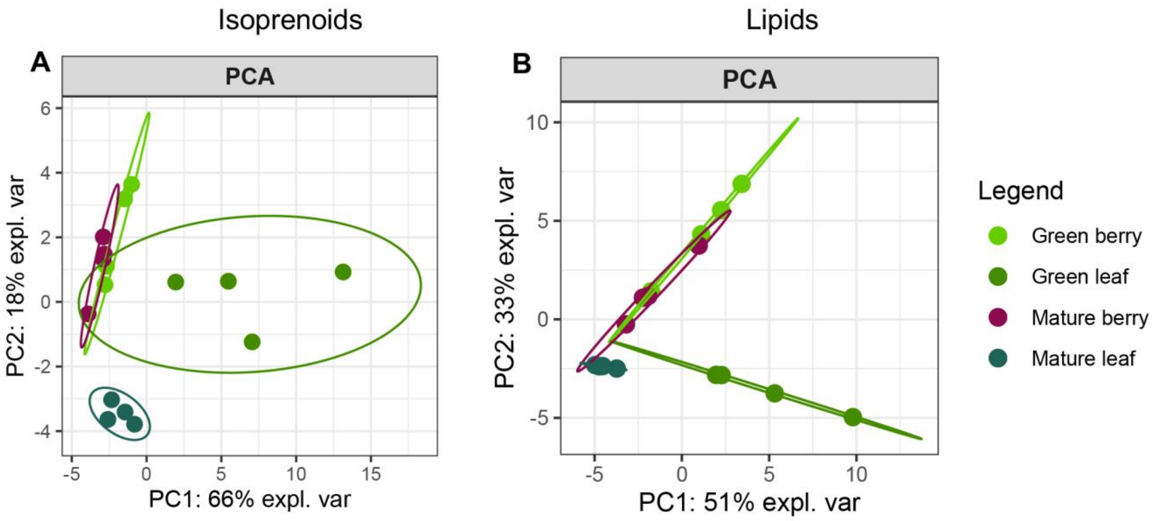


**Supplementary Figure 2.** Targeted metabolomic data from leaf and berry exocarp plastids from cv. “Vinhão”. (**A**) Unsupervised Principal Component Analysis (PCA) of Isoprenoids and (**B**) lipids. Variables in score plot were colored according to the tissue and developmental stage.

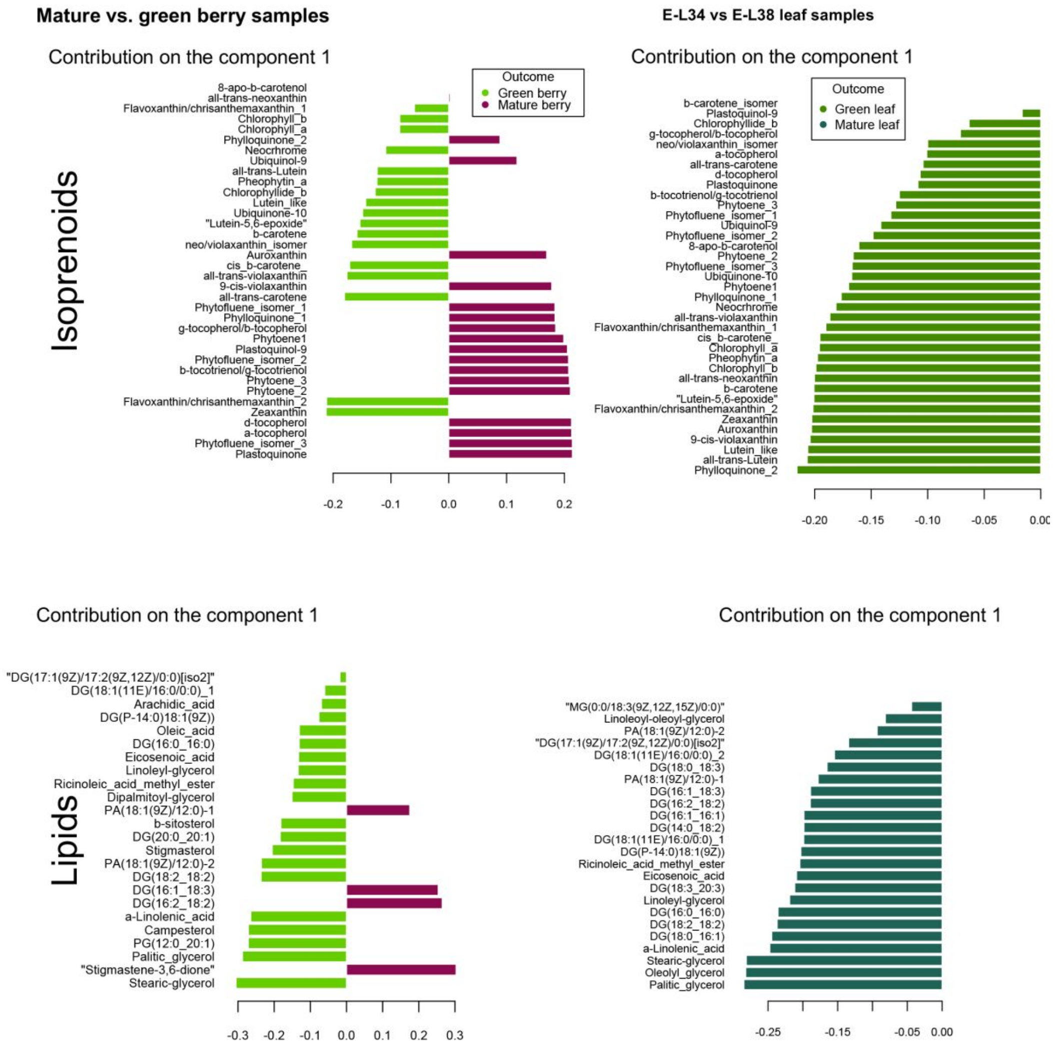


**Supplementary Figure 3**. Plot loadings of PLS-DA analysis of isoprenoids presented in Figure. 3 and lipids presented in Figure. 5.

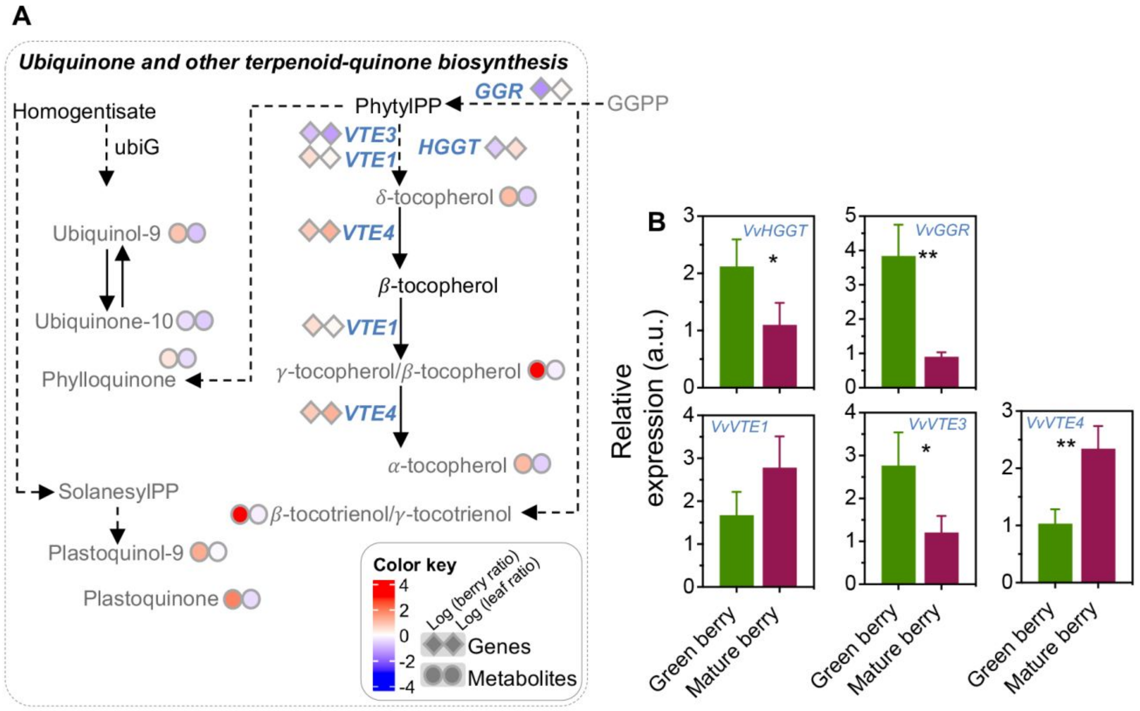


**Supplementary Figure 4**. Metabolites and key transcripts of quinone biosynthesis identified in both purified plastids tissues from the skin of grape berries and leaves at green (E-L 34) and mature (E-L 38) stages of development. (A) Relative log ratio (log2 mature/green) of quinone content and key gene expression in purified plastids and transcripts of key genes from berries and leaves at green and mature stages of development of the red grape berry cv. "Vinhão". Asterisks indicate statistical significance between mature (E-L 38) and green (E-L 34) conditions following the Student's t-test: *P ≤ 0.05; **P ≤ 0.01.

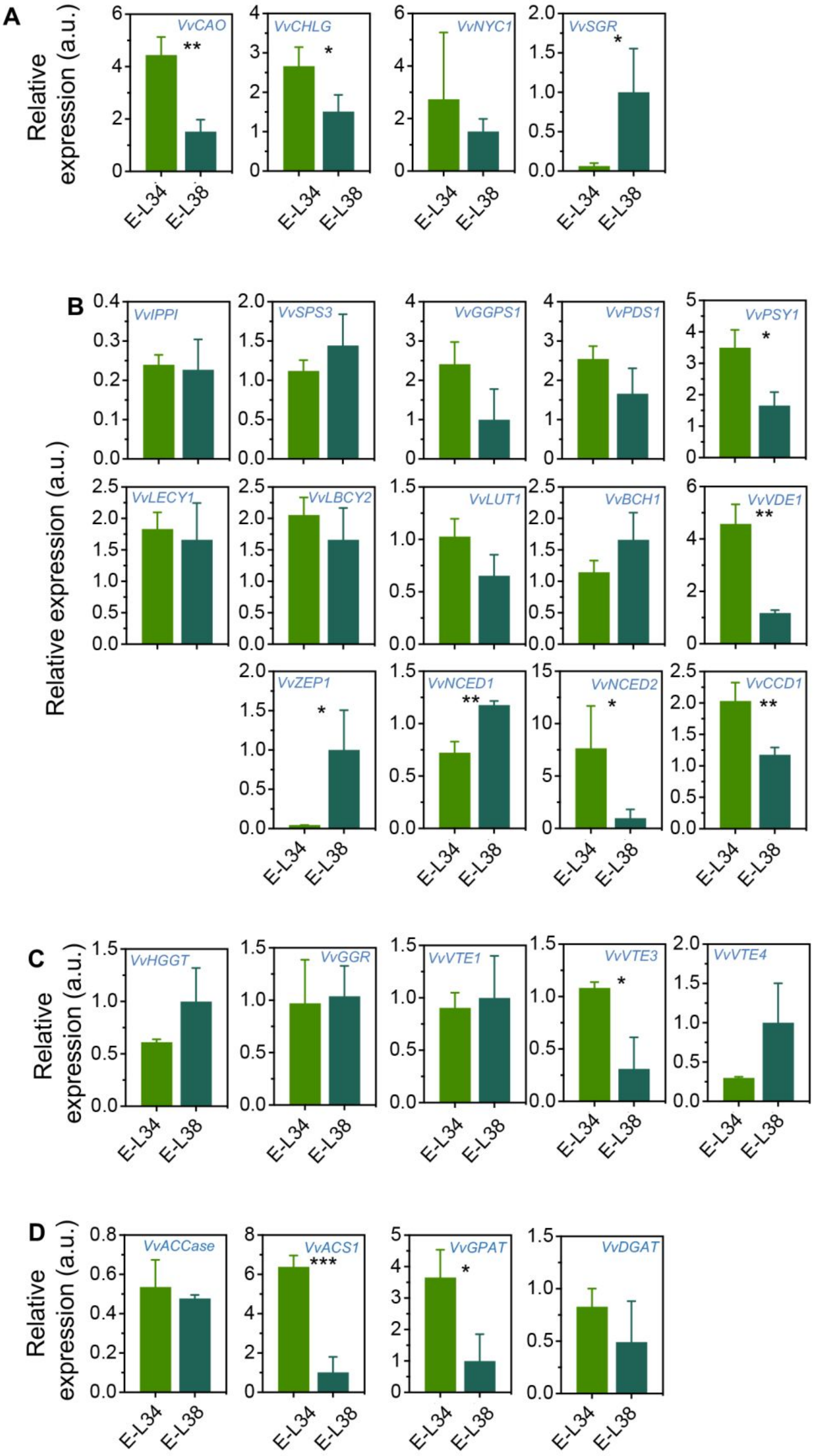


**Supplementary Figure 5.** Relative expression of key genes of grapevine leaves collected at green (E-L 34) and mature (E-L 38) stages of red grape berry cv. "Vinhão", involved in the biosynthesis of (A) chlorophylls, (B) isoprenoids and (C) lipids. Asterisks indicate statistical significance between mature (E-L 38) and green (E-L 34) conditions following the Student's t-test: *P ≤ 0.05; **P ≤ 0.01; ***P ≤ 0.001.

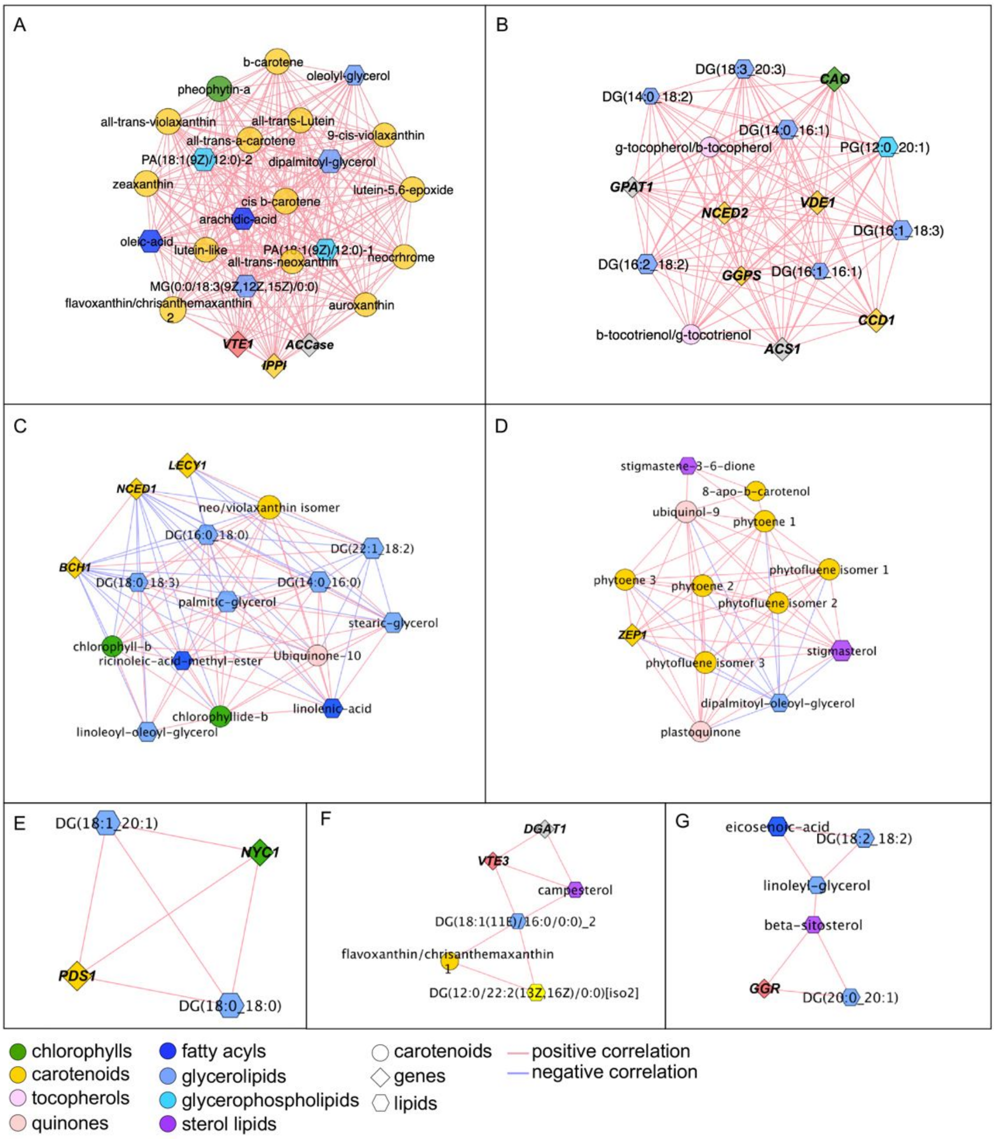


**Supplementary Figure 6.** Subnetworks generated using the MCODE Cytoscape plugin applied to the global correlation network shown in **figure 7**, and ordered according to descending density (A, B, C, D, E, F). Each node represents a transcript (diamonds) or a metabolite (circles-isoprenoids; hexagon-lipids). Lines joining the nodes represent correlations (ρ>|0.90|); positive correlations are shown in red, while negative correlations are shown in blue. Edge thickness is proportional to the respective correlation value (r) and node size is proportional to the correlation node strength (ns =|ρ|). For more details, see *Material and Methods* and Supplementary Table 5.
